# The Decision Tree for Clinical Management of Dentin Hypersensitivity. A Consensus Report

**DOI:** 10.3290/j.ohpd.b2572997

**Published:** 2022-01-20

**Authors:** Gianna Maria Nardi, Silvia Sabatini, Giovanna Acito, Arcangela Colavito, Lorella Chiavistelli, Guglielmo Campus

**Affiliations:** a Professor and Dental Hygienist, Department of Dental and Maxillo-Facial Science, School of Dentistry, Sapienza University of Rome, Rome, Italy; Academy of Advanced Technologies in Oral Hygiene Sciences (ATASIO), Rome, Italy. Contributed to conception and design, critically revised the manuscript, approved final manuscript.; b Lecturer and Dental Hygienist, Academy of Advanced Technologies in Oral Hygiene Sciences (ATASIO), Rome, Italy. Contributed to conception and design, drafted the manuscript, approved final manuscript.; c Dental Hygienist, Academy of Advanced Technologies in Oral Hygiene Sciences (ATASIO), Rome, Italy. Collected completed questionnaires, approved final manuscript.; d Professor, Department of Restorative, Preventive and Pediatric Dentistry, School of Dental Medicine, University of Bern, Switzerland; Department of Medicine, Surgery and Experimental Sciences, School of Dentistry, University of Sassari, Sassari, Italy; Faculty of Dentistry, Sechenov First Moscow State Medical University, Moscow, Russia. Contributed to conception and design, data analysis and interpretation, drafted and critically revised the manuscript, approved final manuscript.

**Keywords:** consensus, dentin hypersensitivity, diagnosis, Italy, prognosis

## Abstract

**Purpose:**

To reach a consensus on a consistent strategy to adopt when screening patients for the clinical management of dentin hypersensitivity.

**Materials and Methods:**

A panel consisting of members of the Advanced Technology in Oral Hygiene Sciences Academy (ATASIO) was formed to start a review process on dentin hypersensitivity (DH) and subsequently elaborate a decision tree to manage DH, from diagnosis to prognosis. The panel employed the RAND in their deliberations. After an initial systematic literature review, it became evident that a consensually validated protocol for the management of patients affected by dentin hypersensitivity has to be considered mandatory by all dental professionals. However, the outcome of the systematic review made it evident that the treatment options to be provided, as well as their prognosis and timing, had never been defined. The panel produced documents that addressed the topic and were subsequently used to generate a questionnaire. A workshop of expert dental professionals was organised to reach consensus on the main steps of the decision tree. Each member completed the questionnaire independently, and then a panel discussion was held to reach a consensus.

**Results:**

A high level of agreement was reached regarding all the items on the questionnaire, and each of the clinical questions formulated was answered. A clinical decision threshold was created.

**Conclusions:**

The dissemination of the information to a wide dental audience should commence upon publication of this consensus document. The authors hope that this consensus will become a model for the development of a dedicated protocol to manage DH.

The Canadian Advisory Board on Dentin Hypersensitivity (DH) of 2003^9^ revealed important knowledge gaps among dentists and dental hygienists regarding aetiology, diagnosis and management of DH. In a total of 542 dental professionals interviewed, 14 knowledge gaps were observed. Half of the respondents considered that differential diagnosis is necessary for DH. 17% of the dentists and 48% of the dental hygienists failed to identify the hydrodynamic theory as a cause of DH, and 64% of dentists and 77% of dental hygienists considered bruxism and malocclusion as triggers of DH. 56% of dentists and 68% of hygienists considered desensitising toothpaste effective in preventing DH, but 31% of dentists and 16% of hygienists did not consider that desensitising toothpastes can relieve DH.

In the following years, several authors tried to estimate the real epidemiological figures,^13,28^ attempt a diagnosis process,^12^ and propose protocols to manage DH.^1,2,10,21,23,30^ Also, a decision tree was designed.^19^ Despite these measures, the scientific literature still provides conflicting information.

In Italy, DH afflicts 45% of the young adult population,^14^ while in Europe, DH prevalence has a wide range from 1.34% to 98%.^32^

The knowledge gaps among dental professionals were confirmed by a more recent survey of Brazilian dentists;^32^ the outcomes confirmed that the main causes of DH were incorrectly considered to be premature contacts. Surprisingly, almost one-third of the respondents did not provide any therapy for managing DH.

This paper provides a synopsis of the available evidence to provide clinical recommendations for the management of dentin hypersensitivity. The aim of this consensus project was to utilise a RAND process^11^ to reach agreement on the adoption of a consistent and effective strategy to manage DH.

## Materials and Methods

Based on these premises and after agreement on the need for this consensus process, a working group of members of the Advanced Technology in Oral Hygiene Sciences Academy (ATASIO) was established in order to start a review process on dentin hypersensitivity (DH) and subsequently elaborate a decision tree to manage DH from diagnosis to prognosis. After completing the review process and elaborating the decision tree, a workshop of expert dental professionals was organised to reach a consensus on the main steps of the decision tree.

**Fig 1:**
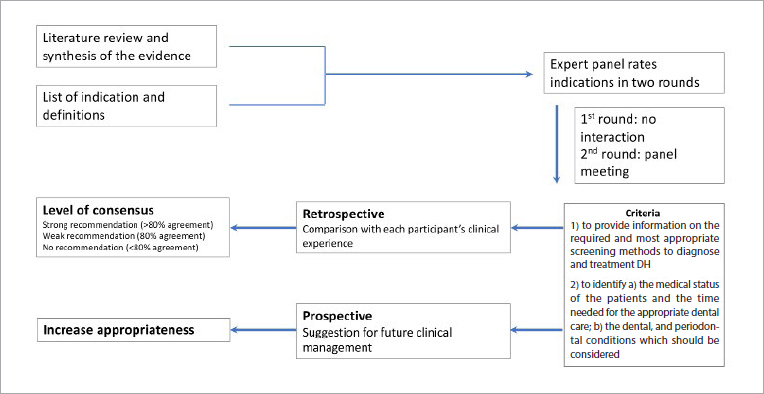
The RAND procedure.

The RAND/UCLA appropriateness method (RAM)^12^ was selected to achieve consensus. One of the current authors (GC) provided methodological expertise for the RAND process. In the subsequent joint e-Delphi workshop, the final wording of the statements was developed, striving to include aspects from the fields of restorative/conservative dentistry, endodontics, periodontology and preventive dentistry. The consensus process was divided into three phases (Fig 1).

### Consensus Procedure

The RAND/UCLA appropriateness method (RAM)^12^ was used to reach consensus (Fig 1). RAND is a modified Delphi method, approved by major institutes,^6^ that was developed to identify the opinion of experts and enable the measurement of the use of clinical procedures. Since consensus does not need to be defined as full agreement among participants, a pre-specified target of 80% agreement was approved.

A systematic review was carried out to evaluate the information available on PubMed (718 papers), Scopus (221 papers) and Web of Science (96 papers) from the inception of each database up to 30th October 2020 using Boolean operators to combine MESH and free-text words. A group of three dental hygienists (GA, LC, AC) and a dentist (GC) carried out the review process. Only clinical trials and systematic reviews were considered eligible. Duplicates, unnecessary papers and off-topic papers were excluded by reading titles and abstracts. The remaining papers (n=67) were fully examined. As the review included a large heterogeneous array of study designs and sources, the results were synthesised using a narrative approach.

The most recent systematic reviews on several aspects of DH^11,17,20^ were selected as the basis for the consensus.

The reviews were coordinated by GC, complying with the purpose to elucidate the following PICO (patient/population/problem, intervention, comparison, outcome) questions:

1. How and when should one intervene in DH?
  - What is the effect of non-invasive/preventive treatment options on DH?
  - What are the success rates of invasive/operative treatment options of DH (e.g. conservative or endodontic treatments)?
2. How should one intervene in patients with DH?

Based on these 3 systematic reviews^11,17,20^ on DH, several structured consensus statements and clinical recommendations were drafted by two authors (GN, SB). After the review process, two others dental hygienists (SS, GN) elaborated 10 statements regarding the definition, aetiology, diagnosis and treatment of DH. The statements were obtained considering only the information and results that were congruent in at least five different articles among those eligible.

### Decision Tree

Using the ten main statements elaborated, a decision tree was constructed by a dental hygienist and a dentist, considering in the results the main steps of diagnosis, treatment and maintenance.

#### Selecting the panel of experts

A panel of experts, selected on the basis of research, academic and practical expertise and level of English language proficiency, from the Advanced Technology in Oral Hygiene Sciences Academy (ATASIO) were chosen. They received the structured consensus statements and clinical recommendations prior to the workshop meeting in Rome, Italy.

#### Consensus panel meeting

At the meeting, each statement was broadly discussed and modified until a consensus was reached. The strength of each recommendation was evaluated by the group and classified as ‘strong,’ ‘moderate,’ or ‘weak’ based on the scientific evidence supporting the statement. Recommendations supported by unequivocal evidence (e.g. many randomised controlled trials) were evaluated as ‘strong.’ Recommendations based on moderate evidence (e.g. high-quality clinical studies, such as randomised controlled trials with similar results) were evaluated as ‘moderate.’ Finally, recommendations based on expert opinion only and those based on weak evidence (e.g. no clinical studies or only low-quality studies or studies with contradicting results) were ranked as ‘weak.’

Based on the discussions at the meeting, this paper was drafted by the consensus panel meeting and sent to the overall group, who commented on it extensively, in 2 rounds. The dental professionals were asked to express their agreement to each statement as follows: the responses to a given statement were scored from 1 (completely disagree) to 10 (completely agree). At least 70% of the responses with a score > 7 was considered as acceptance of the statement by the group, and the results were reported as agreement (i.e. 10 to 8), neutral (i.e. 7 to 4), or disagreement (3 to 1). In addition, the median of all of scores was calculated. An additional field for free-text comments was also available to allow naming the reason for a certain decision or proposals for future modifications.

### Survey

A survey was conducted to assess the agreement of several dental professionals, particularly dental hygienists, with the statements taken from the review process. 216 dental professionals, 178 dental hygienists (82.41%) and 38 (16.59%) dentists were involved in this survey and were asked to answer to a self-administered questionnaire. Ten statements were put forward, regarding definition, aetiology, diagnosis and treatment of DH.

## Results

### Survey

Consensus was established apriori, with 80% agreement among participants. Each statement was discussed by the chairperson of the workshop, then the questionnaire was delivered to each of the 216 dental professionals to be filled out confidentially/anonymously. Each statement below is followed by the percentage of agreement by the dental professionals.

#### Definition

1. Dentin hypersensitivity^7,8,10^ is a short and painful response to an external stimulus – thermal, chemical or tactile – that is applied on the cervical area of the vestibular surface of a tooth. Cervical areas are the most commonly affected areas as reported in literature (>85%)^5^.

#### Aetiology

2. Hydrodynamic theory:^8,22^ the plasma-like biological fluid contained in the dentinal tubules can flow, stimulating the mechanoreceptors of the pulp and consequently causing pain (95.8%).

3. Role of oral biofilm:^16^ oral biofilm accumulation in the cervical area can lead to peri-tubular dentin decalcification with consequent dentinal tubule enlargement, causing DH (69.4%).

#### Diagnosis

4. Interviewing the patient:^10,19^ talking and listening to the patient and asking specific questions promotes better understanding of the patient and is a crucial step toward correctly diagnosing DH (100%).

5. Differential diagnosis:^13^ other oral pathologies, such as caries or periodontal disease, must be excluded before treating a patient for DH (94.4%).

6. Schiff Index collection:^26,27^ the Schiff index is considered the index of choice for monitoring DH.^27^ It is crucial to collect and document the Schiff index score for each tooth before starting any treatment of DH (83.4%).

7. Systemic correlation:^28,29^ DH is detectable in patients who suffer from xerostomia or bulimia, or who take antihypertensive medication (87.5%).

#### Treatment

8. Toothpastes:^18,23^ they are considered the most effective at home therapy to manage DH (56.9%).

9. Fluoride varnishes:^17,21^ the application of fluoride varnishes, also more than once, is considered the most effective professional therapy for managing DH (75.0%).

10. Dental treatment:^18-20^ before proceeding with more invasive therapies, such as conservative or endodontic treatment, it is appropriate to perform a correct diagnosis and be sure that DH is not resolvable with at-home treatment or varnish application (97.20%).

### Decision Tree

**Fig 2:**
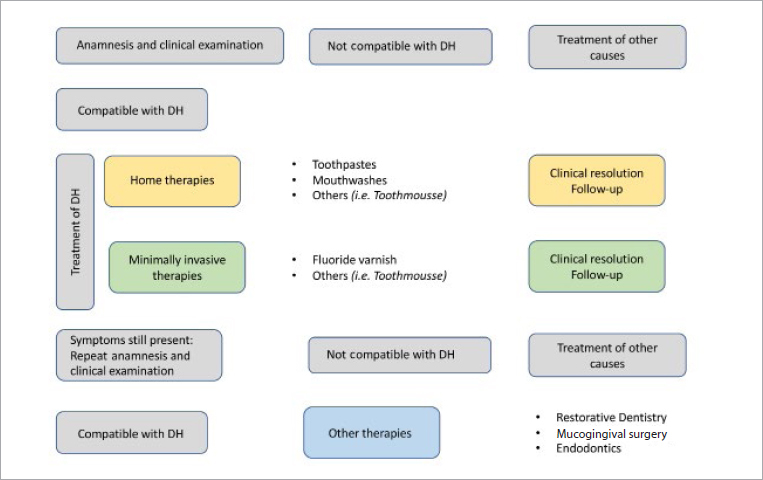
The decision tree.

Considering a patient apparently suffering from DH, the first question to answer is if the symptoms are compatible with DH (Fig 2). If the answer is negative, other causes must be identified and treated. If the symptoms are compatible with DH, therapy for DH can be initiated.^18-20^

To treat DH, the first thing to do is to remove and correct any possible cause or aggravating factors. If the patient shows no more symptoms, we can assign her/him to a follow-up programme. If the patient still has complaints, we can prescribe at-home therapy with toothpastes^18,23,30^ as the first choice, and/or mouthwashes and/or mousse. According to the literature,^4,25^ the most effective substances present in toothpastes are strontium chloride, stannous fluoride and potassium nitrate. More recently, other products have become available, but with only moderate or little evidence to support their efficacy.^2,3,15,24^

At this point, if the therapy is effective, the patient can be enrolled in a follow-up or minimally invasive professional therapy programme if the symptoms persist. The treatments can consist in fluoride varnish application as the first choice,^21^ and/or laser therapyand/or tooth-mousse application. If minimally invasive treatments are effective, the patient can be enrolled in a follow-up programme.

If the patient continues to suffer from DH, a clinical examination should be carried out and anamnesis should be repeated. If any other cause can be discovered, it must be treated and removed. If the symptoms are compatible with DH without any other pathology afflicting the oral cavity, more invasive measures can be considered, such as conservative treatment, mucogengival surgery, or endodontic treatment.^17^

## Discussion

The proposed decision tree on DH was widely accepted by the dental professionals, as demonstrated by the answers to the survey. The small sample of participants can be considered a limitation. On the other hand, the accuracy of the review process and the elaboration of the survey data can be considered a strength.

Compared to the dental professionals interviewed by the Canadian Advisory Board on Dentin Hypersensitivity,^6^ those who answered our survey seemed to be more aware of aetiology and management of DH.

The hydrodynamic theory was widely accepted (95.8%), as was the definition of DH (94.5%). Understanding the mechanisms that trigger DH and being able to define the aetiology and the clinical situation can be considered the right starting point to diagnose properly and manage the problem well.

The professionals involved were unanimously aware (100%) that the interview with the patient is the crucial point, and the best way to begin diagnosing DH. Our results do not permit a conclusion about whether the participants usually screen their patients for DH, but it possible to declare that they know they should start with an interview.

94.4% of them considered that a differential diagnosis for DH is necessary, and were aware that other pathologies should be excluded to properly define a case of DH. 87.5% agreed that considering systemic involvement in the clinical picture was necessary.

Concerning Schiff’s index,^26,27^ the participants underlined the importance of recording it before starting any treatment. The Schiff cold-air sensitivity scale was advocated to assess the subject’s response to a stimulus such as air or evaporation.

Almost all of the dental professionals (97.2%) agreed that diagnosis is crucial before considering invasive therapies.

It is possible to conclude that these dental professionals were correctly informed about the diagnosis process. Concerning the therapies, the main deficit among the respondents was revealed. Despite evidence from several studies,^1,2,23,30^ many dental professionals (43.1%) are not aware of the effectiveness of specific toothpastes in the management of DH. This can be explained by a possible knowledge deficit in terms of toothpaste products, or it may be due to the fact that some dental professionals believe that desensitising toothpastes have low efficacy in daily clinical practice. The use of such toothpaste is completely left to the patient, with poor possibility of control by dental professionals. It can be supposed that a dentist or a dental hygienist may not feel it is adequate to delegate DH management completely to a toothpaste.

The particpants agreed that fluoride varnishes are an effective professional treatment. Although the percentage of agreement with this item was 75% and thus reached consensus level, it is obviously far from the scores given for the diagnosis questions.

All in all, we can state that the 216 dental professionals involved were definitely aware of the definition, aetiology and diagnosis of DH, and sufficiently agreed with the therapies considered effective. The decisional pathway was developed taking into account the statements proceeding from the review process, and the respondents agreed with the statements, so we can conclude that this decision tree is accepted by the clinicians.

## Conclusion

Today, each decision pathway that leads to a choice in dentistry, as in medicine in general, should be carried out based on scientific literature and clinical evidence. The existing gap between scientific literature and clinical practice should be filled. For this reason, it is extremely important that clinical protocols tested and proposed by authors be available and agreed upon by the final recipients: dentists and dental hygienists. A regular revision of the decision tree is advisable in order to guarantee the availability of consistently updated protocols.
